# Awareness of Social Influences on Eating Is Dependent on Familiarity With Imagined Dining Partners and Type of Eating Occasion

**DOI:** 10.3389/fpsyg.2022.841422

**Published:** 2022-04-26

**Authors:** Suzanne Higgs, Ayoub Bouguettaya, Helen Ruddock

**Affiliations:** School of Psychology, University of Birmingham, Birmingham, United Kingdom

**Keywords:** social influences, eating, awareness, social inhibition, social facilitation

## Abstract

When eating with strangers, people tend to eat less than they would when eating alone (social inhibition of eating), whereas they tend to eat more with family and friends (social facilitation of eating). To assess awareness of the social inhibition and facilitation of eating we conducted two online studies (Study 1:N = 481; Study 2:N = 485). In Study 1, participants imagined a dining scenario and indicated whether they would eat the same, more, or less when eating with someone who was more or less familiar to them (friend; family member; acquaintance; stranger) compared with when eating alone. Results from Study 1 indicated that participants imagined eating special meals with friends/family and so, in Study 2, another group of participants made the same predictions as for Study 1, but they did so for special and regular meals. In Study 1, a significant majority of participants said that they would “eat less” with a stranger/acquaintance. A similar proportion of participants said that they would “eat the same” or “eat more” when with a friend/family member and significantly fewer participants said that they would “eat less” with a friend/family member. In Study 2, the majority of participants said that they would “eat less” with a stranger across both special and regular meals. For meals with an acquaintance, the majority of participants reported that they would “eat the same” during regular meals, while for special meals, equal numbers said that they would “eat the same” as would “eat less.” The majority of participants indicated that they would “eat more” during a special meal with a friend/family member. However, for regular meals with a friend/family member, a significant majority of participants said that they would “eat the same.” Hence, participants acknowledge the social inhibition of intake and the social facilitation of eating at special meals, but they are either unwilling or unable to acknowledge that they would eat more with a friend/family member at a regular meal compared with eating alone. Raising awareness that eating with friends/family is associated with greater intake at regular meals may be helpful for individuals who are trying to manage their intake.

## Introduction

The social context in which food is consumed affects what and how much is eaten (Herman et al., 2003; Higgs and Thomas, 2016). For example, people tend to eat less when in the company of strangers compared with dining alone (Vartanian et al., 2007). This social inhibition of intake likely occurs because people try to manage the impression they make on their dining partners (Vartanian, 2015). Most people are motivated to present themselves in a positive light, especially to strangers (Baumeister and Leary, 1995), and one way in which a positive image can be conveyed is *via* amounts of food consumed (Vartanian et al., 2007). This is because the amount of food eaten tends to be associated with personality characteristics, known as consumption stereotypes. For example, there is evidence that people who consume large portions tend to be perceived negatively, whereas those who consume small portions are rated as more attractive, likeable, and moral (Steim and Nemeroff, 1995). Thus, when eating with strangers, people may choose to consume a small portion (less than they would if eating alone) because they think this will convey a positive image.

On the other hand, when eating with friends and family (or otherwise familiar diners), people tend to eat more when than they would when eating alone (de Castro and de Castro, 1989; Ruddock et al., 2019). This phenomenon is known as the social facilitation of eating (de Castro, 1991). Social facilitation has been found to be a robust and large effect that occurs at all meal types, including meals taken with alcohol at the weekend, as well as weekday breakfasts (De Castro et al., 1990; de Castro, 1991). These observations suggest that social facilitation of eating is not an artefact that arises because people are more likely to eat with others during celebrations (at which feasting is part of the social occasion). The precise mechanisms underlying the social facilitation of eating are unclear. However, recent theorising suggests that it may be a hard-wired psychological phenomenon which evolved as a strategy to maximise personal food intake in the context of sharing limited food resources with members of a close social group (Ruddock et al., 2021a).

Little is known about the extent to which people are aware of, and/or willing to acknowledge, social inhibition and facilitation effects on eating. If people are unaware of how their eating is affected by others, then this lack of knowledge has implications for how people manage their consumption. For example, reaching one’s health goals may be difficult if one is unaware of situations that might pose a challenge to reaching those goals. Previous research suggests that when people are asked about what determines how much they eat, they tend to highlight internal factors, such as hunger or “liking” for the food rather than factors such as the portion size provided or the behaviour of others (Vartanian et al., 2008). This is the case even when hunger and liking are unrelated to the actual amount eaten (Vartanian et al., 2008). However, under certain circumstances, some people are willing to acknowledge the influence of external factors, such as the social context of eating, on their consumption (Spanos et al., 2015; Vartanian et al., 2017a). For example, when people believe they have consumed more than they normally would, external influences may be invoked to explain the excessive intake (Vartanian et al., 2017a). Therefore, it may be that people would tend to only acknowledge socially facilitated intake if it is perceived to be in excess of what they would normally eat *alone*. In a recent study, we reported that people serve themselves more in advance of a meal that they know is going to be eaten with a friend, compared with what they serve themselves when they know they are going to be eating alone— suggesting that on some level they may be aware that eating with others is associated with greater intake (Ruddock et al., 2021b). In terms of social inhibition of intake, because impression management rests on knowing that certain behaviours are stereotypically related to the kind of impression one wishes to make (Vartanian et al., 2007), then it might be that people are aware that eating less in company is a deliberate impression-management tactic.

No previous studies have investigated awareness of the social facilitation/social inhibition of intake. Therefore the present studies addressed this gap in the literature. An online methodology was used in which participants were asked to imagine a dining scenario and indicate whether they would eat the same, more, or less when eating with another diner compared to eating alone. Participants were asked about eating with a partner who differed in the degree of familiarity: friend; family member; acquaintance; and stranger. This was because social facilitation is more like to be observed with a familiar dining companion and social inhibition is more likely to be observed with an unfamiliar companion and so we wanted to capture a range of social relations.

## Method (Study 1)

### Participants

We aimed to recruit 500 participants (approximately equal numbers of males and females) *via* the survey platform Prolific. The sample size was calculated to provide 85% power to detect small effect sizes (*w* = 0.15) using Chi-Square tests (degrees of freedom = 2, significance level *a* = 0.05). Participants were led to believe that the aim of the study was to examine the relationship between mood, context, and attitudes toward eating. Participants were informed about the eligibility criteria in the participant information sheet: (1) Aged 18 and over, (2) fluent in English, (3) no prior diagnosis of disordered eating, and (4) had not taken part in any of our previous similar studies but had already been pre-screened by the survey platform for eligibility. The study protocol was approved by the University of Birmingham’s Research Ethics Committee and participants indicated their informed consent to take part. The study method, hypotheses, and analysis plan were preregistered on the Open Science Framework website.^1^

### Measures

#### Awareness Assessment

To assess awareness of social influences on eating, participants were asked: “Compared to how much you’d eat when you’re alone, please indicate how much you would eat when with (friends/family/strangers/acquaintances).” Acquaintance was defined for the participants as “someone you know slightly but not well.” Responses were provided on a 5-point Likert scale (“Eat a lot less,” “Eat slightly less,” “Eat the same,” “Eat slightly more,” “Eat a lot more”). To disguise the aims of the study, participants were also asked about how much they eat when feeling anxious, angry, sad, and happy (relative to when feeling neutral) (data not reported). Questions were presented in random order across participants. Participants completed all questions (8 in total).

Participants were then asked to state, using a free-text response format, *why* they had responded the way they had to each awareness question (i.e., “why did you say that you would eat more/less/the same when with strangers/family/friends/acquaintances, compared to when alone?”).

#### Appetite and Mood Measures

Current levels of “hunger” and “fullness” were assessed using 100 point Visual Analogue Scales (VAS). Each scale was anchored by “Not at all” (0) to “Extremely” (100). An overall “Appetite” rating was calculated by averaging across “hunger” and the inverse of “fullness” ratings. To maximise the believability of the cover story, 100 point VAS ratings were also obtained for a variety of mood states (angry, sad, subdued, agitated, neutral, excited, anxious, happy, calm, bored).

#### Eating Behaviour Traits

Participants completed two eating trait questionnaires to allow us to characterise our population.

##### Social Eating Behaviour

Responsivity to social eating cues was assessed using the Social Eating Scale (Spanos et al., 2014). The scale is modelled on the External Eating subscale of the Dutch Eating Behaviour Questionnaire (DEBQ, Van Strien et al., 1986) which comprises two questions related to social influences on food intake (e.g., If you see others eating, do you also have the desire to eat?). Spanos et al. (2014) added a further four items to create the Social Eating Scale (e.g., If the person you are eating with eats a lot, do you also eat a lot?). Responses to each item were provided on a 5-point scale ranging from “Never” to “Very often.” The six items were averaged to provide a score for each participant. Higher scores indicate greater tendency to eat in response to social influences.

Frequency of social eating was assessed using the following item: “In an average week, how often do you eat a meal with at least one other person?”. Response options ranged from “Not at all” to “More than seven times a week.”

##### Three-Factor Eating Questionnaire (TFEQ-18R)

The TFEQ-18R (Karlsson et al., 2000) comprises three subscales: (1) The cognitive restraint subscale consists of six items which assess the tendency to restrict intake in order to control weight (e.g., “I deliberately take small helpings as a means of controlling my weight”); (2) The emotional eating subscale consists of three items and measures the tendency to eat in response to negative moods (e.g., “When I feel blue, I often overeat”); (3) The uncontrolled eating subscale has nine items and assesses perceptions of control over food intake (e.g., “Sometimes when I start eating I just can’t seem to stop”). Total scores were calculated for each subscale. Higher scores indicate greater dietary restraint, emotional eating, or uncontrolled eating.

### Procedure

The questionnaire was presented using Qualtrics software. After providing informed consent, participants completed questionnaires in the following order: (1) Appetite VAS; (2) Mood VAS; (3) Awareness assessment; (4) Appetite VAS; (5) Mood VAS; (6) Social Eating Scale; (7) Social eating frequency; (8) TFEQ-18R. Participants then indicated their gender, age, ethnicity, and stated whether or not they were currently a student. Due to the online nature of the study height and weight were self-reported (to calculate BMI). Self-report tends to underestimate weight and overestimate height (Gorber et al., 2007), but as BMI was not a primary outcome, self-reported values were deemed to be acceptable to describe the population. To check that participants were paying attention during the study, two “attention check” items were incorporated within the questionnaires, in which participants were asked to select a specific response (e.g., “please select Definitely True”). Participants who failed both attention check questions were removed from subsequent analyses. To assess the presence of demand characteristics, participants were asked to indicate what they thought were the aims of the study were, and what they believe we had predicted (open text response). Participants were then presented with a debrief which explained the true aims of the study.

### Data Analysis

Responses to awareness questions were coded into one of three categories: (1) “eat less” (i.e., “eat a lot less”/”eat slightly less”), (2) “eat the same,” or (3) “eat more” (i.e., “eat a lot more”/”eat slightly more”). Chi-square tests of goodness-of-fit were performed to test the null hypothesis that, for each scenario type, cell frequencies would be distributed equally across “eat less,” “eat the same,” and “eat more” responses. Where significant differences were identified, pair-wise comparisons were conducted between each of the three response types (e.g., eat less vs. eat the same; eat more vs. eat the same; eat less vs. eat more) to identify which cell frequencies differed from each other. Statistical analyses were conducted using SPSS version 27.0.

Participants’ free-text responses to the follow-up questions (i.e., *why* they had responded the way they had to each awareness question) were thematically analysed by assigning codes to each response and then grouping codes into overarching themes (Braun and Clarke, 2006).

## Results (Study 1)

### Participants

A total of 486 participants completed the study. Participants who failed both attention check questions (*n* = 3), or who were identified as outliers with regards to the amount of time taken to complete the questionnaire (i.e., *z*-scores of above 3 or below −3) (*n* = 2), were removed prior to analyses. Analyses were conducted on 481 participants (males = 229; females = 248; non-binary = 4) (see Table 1 for participant characteristics). The majority of participants (*n* = 279) reported a BMI less than 25 kg/m^2^ (i.e., non-overweight), and 188 participants reported a BMI above 25 kg/m^2^ (i.e., having overweight/obesity). The majority of participants were Caucasian (*n* = 393), and just over one-third of participants were students (*n* = 174). Sixteen participants guessed the general area under investigation (social influences on eating) but none guessed that the specific aim of the study was about awareness of these effects. Removing these participants did not affect the overall findings and so these data were retained in the following analyses.

**TABLE 1:** Participant characteristics Study 1.

	Mean (standard deviation)
BMI (kg/m^2^)	24.89 (5.01)
Age (years)	30.16 (10.30)
TFEQ-dietary restraint	13.24 (3.83)
TFEQ-uncontrolled eating	19.22 (5.18)
TFEQ-emotional eating	6.82 (2.71)
Social eating scale	2.52 (0.65)
Appetite score (0–100)	47.43 (27.20)

### Main Analysis

For all four social scenarios, responses to the awareness questions were not equally distributed across “eat less,” “eat the same,” and “eat more” options (Table 2). Follow-up comparisons showed that, for the friend and family scenarios, significantly more participants said that they would “eat the same” or “eat more,” than would “eat less” (all *p*s < 0.001). Participants were equally likely to say that they would “eat more” or “eat the same” when eating with a friend or a family member.

**TABLE 2:** Response frequency (*n*) for each scenario Study 1.

	Eat less	Eat the same	Eat more	X^2^ Statistic
Friends	93*^a^*	186*^b^*	202*^b^*	X^2^(2) = 43.21, *p* < 0.001
Family	42*^a^*	223*^b^*	216*^b^*	X^2^(2) = 131.16, *p* < 0.001
Strangers	315*^a^*	143*^b^*	23*^c^*	X^2^(2) = 268.71, *p* < 0.001
Acquaintances	282*^a^*	167*^b^*	32*^c^*	X^2^(2) = 195.32, *p* < 0.001

*Different letters denote significant pair-wise comparisons.*

For the strangers and acquaintance scenarios, significantly more participants said that they would “eat less” than would “eat the same” or “eat more” (*p*s < 0.001). Participants were also significantly less likely to say that they would “eat more” than would “eat the same” in strangers and acquaintance scenarios (*p*s < 0.001).

### Reasons Given for Responses

Of those who said that they would “eat more” when with friends or family, a substantial proportion of participants indicated that they had imagined eating special meals with friends/family in which tastier foods are available (Friends scenario: 31%; Family scenario: 20%). These responses included statements such as “Because I am dining out,” “With friends I tend to eat fast food.” Meanwhile, of those who said that they would “eat less” when with friends and family, five percent and 12 percent, respectively, had associated eating with family and friends with the cost of eating or the requirements around eating at home, like sharing food [e.g., “I do not want my friends to blame me for having a high bill at the end of the (meal)” “I have a big family, so I would eat slightly less than I eat when I am alone, so that the food goes further and can feed more people in my family.”].

Of those who said that they would “eat more” when with strangers or acquaintances, 22 percent and 13 percent, respectively, responded this way because they associated meals with strangers/acquaintances as dependent on norms within restaurants or events [e.g., “because eating out at restaurants is awes(some) “I would be eating out with acquaintances. I tend to eat more when I’m eating out”]. Conversely, 1 percent and 2 percent of participants who said that they would “eat less” with strangers and acquaintances, respectively, responded this way because they associated these meals with special events or unique variables associated with context of eating out of home (e.g., “Usually it’s when I eat out of home, therefore I eat less” “different setting from normal, everyday dining situation” “cheaper”).

## Interim Discussion

In Study 1, we found that significantly more participants said that they would “eat more” or “eat the same” when eating with a family member or friend than said they would eat less. When thinking about how much they would eat with a stranger or acquaintance, significantly more participants said that they would eat less than when eating alone than said they would eat more or the same. These results suggest that participants are aware of social inhibition effects when eating with strangers or acquaintances. In addition, some participants appeared to be aware of social facilitation effects because equal numbers said they would eat more as said they would eat the same when eating with a friend/family member. Analyses of qualitative responses suggested that some participants were imagining eating out at special/celebratory meals when thinking about eating with friends/family, which suggests that they may have reported eating more, not just because they were imagining a social eating scenario versus alone, but also because they were imagining eating different types of meals in a different eating context (e.g., eating indulgent foods in a restaurant versus eating a regular meal at home). These potential confounds were addressed in Study 2, in which we aimed to replicate the findings of Study 1, but also control for the different types of meals/contexts by asking participants to imagine both “special” or “regular” meals eaten either socially or alone.

## Method (Study 2)

### Participants

As for Study 1, we aimed to recruit 500 participants (approximately equal numbers of males and females) *via* the online survey platform, Prolific. Participants were led to believe that the study aimed to examine the relationship between mood, context, and attitudes toward eating. Participants were informed about the eligibility criteria in the participant information sheet: (1) Aged 18 and over, (2) fluent in English, (3) no prior diagnosis of disordered eating, and (4) had not taken part in any of our previous similar studies but had already been pre-screened by the survey platform for eligibility. The study protocol was approved by the University of Birmingham’s Research Ethics Committee and participants indicated their informed consent to take part. The study method and analysis plan were preregistered on the Open Science Framework website.^2^

### Measures

#### Awareness Assessment

As in Study 1, awareness of social influences on eating was assessed using a series of questions in which participants indicated how much they would eat when dining with a friend, a family member, a stranger, or an acquaintance, relative to when dining alone. To control for the *type* of meal eaten, participants were asked how much they would eat during a “regular” meal (defined as an everyday, ordinary meal) and a “special” meal (defined as a celebratory occasion, party) consumed with each type of co-eater [i.e., “Compared to how much you’d eat when you’re alone, please indicate how much you would eat when eating (a special meal/a regular meal) with (a friend/a family member/a stranger/an acquaintance)”]. To disguise the aim of the study, participants were also asked how much they would eat when feeling “sad” and “happy,” relative to when feeling neutral (data not reported). Responses to each awareness question were provided on a 5-point Likert scale with the following options: “Eat a lot less,” “Eat slightly less,” “Eat the same,” “Eat slightly more,” or “Eat a lot more.” The order in which “regular” and “special” meal scenarios were presented was counterbalanced across participants; approximately half of participants completed questions about “regular” meals first, and half-completed questions about “special” meals first. Questions referring to each type of co-eater (i.e., Friend/Family/Stranger/Acquaintance) were presented in random order. Participants competed all questions (10 in total).

##### Awareness Assessment: Follow-Up Questions

Follow-up questions were included to examine whether participants had imagined eating different types of food, or within different contexts, when completing the awareness assessment (data not reported). All other measures and overall procedure remained the same as in Study 1.

### Data Analysis

Responses to awareness questions were analysed using Chi-Square tests of goodness-of-fit and *post hoc* comparisons, as described in Study 1. Participants’ free-text responses to the follow-up questions (i.e., *why* they had responded the way they had to each awareness question) were thematically analysed.

## Results (Study 2)

### Participants

A total of 490 participants completed the study. Data from five participants were removed because they were identified as outliers for the time taken to complete the study (*z* score ≥ 3). The remaining sample comprised of 237 males, 242 females, six non-binary (participant characteristics are provided in Table 3). Almost one-third of the sample (*n* = 152) were classified as having overweight or obesity (BMI > 25 kg/m^2^). The majority of participants were Caucasian (*n* = 403), and approximately half were students (*n* = 234). Thirty- nine participants guessed the general area under investigation (social influences on eating) but none guessed that the specific aim of the study was about awareness of these effects. Removing these participants did not affect the overall findings and so these data were retained in the following analyses.

**TABLE 3:** Participant characteristics Study 2.

	Mean (standard deviation)
BMI (kg/m^2^)	24.18 (4.61)
Age (years)	27.66 (9.49)
TFEQ-dietary restraint	12.88 (3.73)
TFEQ-uncontrolled eating	19.05 (4.93)
TFEQ-emotional eating	6.67 (2.85)
Social eating scale	2.57 (0.61)

### Main Analysis

As shown in Table 4, significantly more participants indicated that they would “eat more” when eating a special meal with a friend or family member, than would “eat less” or “eat the same.” However, for regular meals eaten with a friend or family member, a significant majority of participants indicated that they would “eat the same.”

**TABLE 4:** Response frequency (*n*) for each scenario Study 2.

		Eat less	Eat the same	Eat more	X^2^ Statistic
Special meals	Friends	73*^a^*	144*^b^*	268*^c^*	X^2^ = 120.50, *p* < 0.001
	Family	34*^a^*	136*^b^*	315*^c^*	X^2^(2) = 250.32, *p* < 0.001
	Strangers	238*^a^*	168*^b^*	79*^c^*	X^2^(2) = 78.56, *p* < 0.001
	Acquaintances	197*^a^*	182*^a^*	106*^b^*	X^2^(2) = 29.45, *p* < 0.001
Regular meals	Friends	73*^a^*	292*^b^*	120*^c^*	X^2^(2) = 164.44, *p* < 0.001
	Family	49*^a^*	318*^b^*	118*^c^*	X^2^(2) = 241.49, *p* < 0.001
	Strangers	262*^a^*	209*^b^*	13*^c^*	X^2^(2) = 213.28, *p* < 0.001
	Acquaintances	205*^a^*	252*^b^*	28*^c^*	X^2^(2) = 172.61, *p* < 0.001

*Different letters denote significant pair-wise comparisons (all ps < 0.001).*

For stranger scenarios (i.e., regular/special), significantly more participants said that they would “eat less,” than would “eat the same” or “eat more.” However, for acquaintance scenarios, a significant majority of participants said that they would “eat the same” during regular meals, and participants were equally likely to say that they “eat less” and “eat the same” during *special* meals with acquaintances.

### Reasons for Responses

For all strangers and acquaintances scenarios (i.e., regular and special meals), a large proportion of participants who said that they would “eat less” did so because they would not feel comfortable eating with unfamiliar people (e.g., “Sometimes I feel a little awkward when eating with an acquaintance”) (Strangers/Special = 39%; Strangers/regular = 38%; Acquaintances/Special = 38%; Acquaintances/regular = 39%), or to portray favourable impressions of themselves (“It’s a stranger so I have to…make a good impression”) (Strangers/Special = 21%; Strangers/regular = 22%; Acquaintances/Special = 22%; Acquaintances/regular = 30%).

For special meals eaten with friends and family, the majority of participants who said that they would “eat more” did so because they “feel totally comfortable at that situation” (Family = 16%; Friends = 10%), or because the meal would be more enjoyable (Family = 10%; Friends = 19%) (e.g., “With friends I’m always happier and enjoy food a lot more”). The majority of participants said that they would “eat the same” during regular meals consumed with friends and family out of habit or because “it’s a normal meal” (Family = 25%; Friends = 17%).

## Discussion

In the present studies, we asked participants to indicate whether they would eat the same, more or less when eating with a partner, relative to what they would consume when eating alone. Scenarios including co-eaters who differed in the closeness of their relationship to the participants (a friend, family member, acquaintance or stranger) and different types of meals (special versus regular) were imagined. Our aim was to understand more about people’s awareness of the phenomenon of social inhibition intake, which occurs when people eat with others whom they do not know very well, and the social facilitation of intake, which occurs when eating with people we know e.g., a friend or family member. Across both studies, we found that people said they would eat less when eating with a stranger than they would when eating alone, regardless of meal type. After imagining eating with a friend/family member/acquaintance, participants responded that the amount they would eat was dependent on the type of meal presented in the scenario. People said they would eat more at a special meal with a friend/family member and would eat less with an acquaintance. However, when asked about a regular meal, participants said they would eat the same as they would when eating alone when eating with a friend/family member/acquaintance. These data suggest that for some social situations, there is a mismatch between how people think they behave and how they actually behave. People appear to be aware that they are likely to inhibit intake in the company of a stranger regardless of the type of meal and are likely to eat more at special meals in the company of a friend/family member. However, they are either unaware or unwilling to acknowledge that eating a regular meal with a friend/family member is associated with greater intake than when eating alone.

Previous research has found that people are generally unwilling to acknowledge social influences on eating, preferring instead to explain their intake in terms of internal factors such as hunger and taste (Vartanian et al., 2008). However, previous research has suggested that in some situations people are willing to say that their intake is influenced by external factors, including social influences (Vartanian et al., 2017a). Other research has reported predictions about how much would be consumed in scenarios that combined conflicting internal and external cues were influenced by inhibitory external cues (Vartanian et al., 2017b). When participants were asked to think about a scenario in which they were meeting a friend at a café in a hungry state (facilitating internal cue) but their friend eats only a small amount of food (inhibiting external cue), estimates of how much would be eaten was the average of what was predicted for the effect of the cues separately, indicating that both the internal and external cues had influenced the predictions. Spanos et al. (2015) further reported that while social influences on eating are not generally regarded as appropriate explanations for how much one eats, there is a positive relationship between how appropriate a person considers social influences on intake to be and how willing they are to acknowledge these influences. In other words, participants are more likely to acknowledge social influences if they think that social influences are an appropriate way of explaining their behaviour (Spanos et al., 2015). In the present studies we found that participants were willing to acknowledge the inhibitory effect of eating with a stranger. Inhibiting intake in the presence of strangers may be regarded as appropriate because it is based on a shared understanding that eating lightly conveys a positive impression on others (Vartanian et al., 2007). Taken together, these results suggest that social influences on eating may be acknowledged when they are associated with reduced intake (are inhibitory) and are perceived as an appropriate explanation for behaviour.

We also found that participants acknowledged that they would eat more at special meals with friends and family than they would when they eat alone. In this scenario, there are likely multiple cues that are driving predictions about what will be consumed relative to eating alone. For example, celebratory meals may be associated with eating tastier food, and the tastier the food the more food is predicted to be consumed (Vartanian et al., 2017c). Indeed, participants reported that they would eat more at a celebratory meal with friends and family because it would be more enjoyable. Furthermore, there is some evidence that social facilitation effects while eating with friends may be stronger when it comes to high caloric, enjoyable foods (such as cake) which tend to be eaten only at special meals (Clendenen et al., 1994; Hetherington et al., 2006). Therefore, it is perhaps not surprising that participants said they would eat more at special meals with family and friends because the effect is particularly large for these meals— and therefore, more apparent to people in those circumstances. In the present work, it is not possible to separate the influence of the type of meal imagined from the effect of social context on predictions about intake, but it is possible that in addition to characteristics of the food driving predictions about intake, the presence of another person is acknowledged as a way of explaining excessive intake at celebratory meals. This suggestion is in line with other evidence that external influences on eating may be acknowledged if they allow people to explain why they ate more than they would normally would (Vartanian et al., 2017a,c).

Participants in Study 2 said that they thought they would eat the same when eating a regular meal with a friend/family member as they would when eating alone. Social facilitation of intake has been found to occur for all types of everyday meals (breakfast, lunch, dinner) and not just celebratory types of meals (De Castro et al., 1990) and across a range of study designs including lab-based and observational studies (Ruddock et al., 2019). Hence, we have evidence that people are either unwilling or unable to acknowledge that regular meal consumption in the presence of a friend/family member is associated with increased intake relative to solo dining. It is possible that people are aware that they eat more at regular meals with a friend/family member but are reluctant to admit that their intake is influenced by the social context because they do not wish to appear to have a lack of control over their own behaviour (Burger, 1987). Such motivated denial of social influences on eating has been observed previously. Spanos et al. (2014) found that participants were willing to report that the eating behaviour of others was socially influenced at the same time as refusing to acknowledge that their own behaviour was influenced in this way. These data suggest that people may be aware of social influences on eating but are just not willing to admit that they themselves succumb to these influences; this is possibly due to the general belief that eating outside of hunger is considered to be “overeating” by the general public—meaning admitting that if one eats outside of being hungry but rather due to the situation, this might require participants to admit to themselves that they are overeating (Long et al., 2020). Further work is required to unpack whether people are unaware of the social facilitation of eating that occurs at regular meals or whether they are just not willing to acknowledge it. Future work could compare predictions about a participant’s own intake with predictions made about the intake of others to test this assumption. If participants are willing to acknowledge that others (but not themselves) eat more at regular meals with friends and family then this would suggest that they are aware of the social facilitation of intake at regular meals but are motivated to deny it for themselves.

Participants were equally likely to say that they would “eat less” and “eat the same” during special meals with acquaintances, but for regular meals with acquaintances intake was predicted to be the same as when eating alone. This may be because in the special meal scenario acquaintances were treated more similarly to strangers and responses were more influenced by awareness of impression management. In the regular meal scenario, acquaintances may have been treated more similarly to friends/family, perhaps because it is plausible that a regular meal would be taken with someone who is reasonably familiar.

We used an online study to assess awareness of social facilitation and social inhibition of intake. The imagination task required participant to think about how they would behave in different scenarios but it may be that different answers would be provided if participants were asked the same question but in an eating context e.g., just before a meal when food related cues are salient. Future studies could build on the present results to examine whether awareness of social facilitation and social inhibition of intake is increased when people are in a situation when they are just about to consume a meal with either friends/family/acquaintances/strangers. Another point that could be addressed in future studies is whether some people are more willing to report being aware of social inhibition and social facilitation on effects than are others. It is possible that people who are more aware of social influences on behaviour in general may be more likely to acknowledge that they eat more at regular meals with friends/family for example. In addition, it would be fruitful to examine whether individuals who report being unaware of social facilitation effect demonstrate the phenomenon in the laboratory.

The present results further our understanding of the conditions under which people acknowledge social influences on eating but they also have some practical implications. Failure to acknowledge the social facilitation of eating at regular meals with familiar others may pose a challenge for people who are trying to manage their intake because it may result in people eating more than they intend but not knowing why. If overeating is attributed to internal factors rather than to social factors, this may result a sense of personal failure with associated negative consequences. Therefore, enhancing awareness of the social facilitation of eating could enable people to develop strategies to mitigate the potential for overeating. One strategy might be to pre-serve a fixed portion size before a social meal rather than serve family style during a meal (Ruddock et al., 2021b). Greater awareness of social facilitation of eating may also mean that social eating could be used more widely to increase the food intake of undernourished populations e.g., elderly people with reduced appetite. Here there is potential for use of technology to connect remote diners in ways that might recreate the effects of dining in person (Spence et al., 2019). New developments in remote commensality systems are enabling better interactions between diners but further research is required to assess whether or not these systems provide the same benefits of in person eating (Niewiadomski et al., 2019).

In summary, the results from the present studies show that under some circumstances people are prepared to acknowledge that they eat differently with a companion relative to when they eat alone. People appear to be aware of social inhibition of intake when eating with a stranger and of social facilitation of intake when eating with a friend/family member at a special eating occasion, but they are either unwilling or unable to acknowledge eating more with a friend/family member at a regular meal. This is despite the fact that data from other studies indicates that the social facilitation of eating at regular meals is a robust phenomenon that has been observed in both lab-based and observational studies (Ruddock et al., 2019). Hence, we demonstrate a mismatch between people’s self-perceptions when it comes to eating with a familiar other at regular meals, versus what they say about how they behave. This study indicates there appears to be a lack of awareness of a powerful driver of intake: the presence of others on our food consumption.

## Data Availability Statement

The original contributions presented in the study are included in the article/supplementary material, further inquiries can be directed to the corresponding author.

## Ethics Statement

The studies involving human participants were reviewed and approved by the University of Birmingham Ethical Review Committee. The patients/participants provided their written informed consent to participate in this study.

## Author Contributions

HR performed the data collection and analyses. HR and SH drafted the manuscript. AB provided the critical revisions. All authors contributed to the study design and approved the final version of the manuscript for submission.

## Conflict of Interest

The authors declare that the research was conducted in the absence of any commercial or financial relationships that could be construed as a potential conflict of interest.

## Publisher’s Note

All claims expressed in this article are solely those of the authors and do not necessarily represent those of their affiliated organizations, or those of the publisher, the editors and the reviewers. Any product that may be evaluated in this article, or claim that may be made by its manufacturer, is not guaranteed or endorsed by the publisher.

## Footnotes

1
https://osf.io/esmuw

2
https://osf.io/rjp26
